# *Fusarium* Species Associated with Spikes and Grains of Cereal Crops in the Volga Region: Virulence and Toxin-Producing Potential

**DOI:** 10.3390/jof11120841

**Published:** 2025-11-27

**Authors:** Inna B. Chastukhina, Egor A. Ryazanov, Sergey N. Ponomarev, Irina O. Ivanova, Svetlana Y. Pavlova, Ildar T. Sakhabutdinov, Elena V. Osipova, Mira L. Ponomareva, Vladimir Y. Gorshkov

**Affiliations:** 1 Federal Research Center “Kazan Scientific Center of the Russian Academy of Sciences”, 420111 Kazan, Russiasmponomarev@yandex.ru (M.L.P.); 2 Institute of Fundamental Medicine and Biology, Kazan Federal University, 420008 Kazan, Russia

**Keywords:** Fusarium head blight, mycotoxins, intraspecific variability of *Fusarium* spp., virulence, plant infectious diseases

## Abstract

Fusarium head blight (FHB) is a major threat to cereal crops, causing yield losses and mycotoxin contamination. This study investigated *Fusarium* species associated with spikes and grains of cereals in the Volga region, focusing on species diversity, virulence, and mycotoxin production. *F. sporotrichioides*, *F. avenaceum*, and *F. poae* were the most prevalent species isolated from wheat, rye, barley, triticale, and stored grains in the Volga region. Individual strains of *F. culmorum* and *F. graminearum* were also identified. High intraspecific variability in virulence was observed for the first time within *F. sporotrichioides* and *F. poae* species, and highly virulent strains were identified for the first time within these species. Not only symptomatic but also asymptomatic (weakly expressed) infections caused by *F. sporotrichioides* were shown to be associated with the accumulation of high levels of T-2 toxin in the grains of infected plants. *F. sporotrichioides* strains were first demonstrated to exhibit intraspecific variability in zearalenone-producing potential. A *F. graminearum* strain possessing the nivalenol chemotype was first identified in Russia. The study highlights the diversity of the regional FHB pathocomplex and the risks it poses to grain safety.

## 1. Introduction

*Fusarium* species are among the most harmful and extensively studied phytopathogens of agricultural crops [1,2,3,4]. Many species within this genus cause Fusarium head blight (FHB), which is considered one of the most devastating diseases affecting cereal crops worldwide [5]. FHB is associated with bleaching and shrinkage of spikelets and kernels, which are frequently covered with sporodochia and mycelium, leading to poor grain quality and yield losses of up to 70–80% in susceptible cultivars [2,6,7]. Most importantly, during plant colonization, *Fusarium* species can synthesize a wide array of mycotoxins hazardous to human health, contaminating up to 60–80% of the crop yield [8,9,10,11,12]. A particular problem is that *Fusarium* mycotoxins can accumulate during asymptomatic infection, when no FHB symptoms are manifested [1,4,7]; therefore, mycotoxin control of the produced grain is essential to prevent potential threats to consumers and agricultural animals.

Among the most well-known FHB causal agents are *F. graminearum* Schwabe, *F. culmorum* (W. G. Smith) Sacc., *F. cerealis* Burgess, Nelson, Toussoun, *F. asiaticum* O’Donnell, Aoki, Kistler and Geiser, *F. verticillioides* (Sacc.) Nirenberg., *F. avenaceum* (Fr.) Sacc., *F. sporotrichioides* Sherb., *F. poae* (Peck.) Wollenw., *F. acuminatum* Ellis and Everh., *F. equiseti* (Corda) Sacc., *F. langsethiae* Torp and Nirenberg, and *F. tricinctum* (Corda) Sacc. [13,14,15]. These species vary in virulence as well as in the main mycotoxins they synthesize: deoxynivalenol (DON) (*F. graminearum*, *F. culmorum*, *F. asiaticum*), nivalenol (NIV) (*F. graminearum*, *F. culmorum*, *F. cerealis*, *F. poae*, *F. asiaticum*), T-2 and HT-2 toxins (*F. langsethiae*, *F. poae*, *F. sporotrichioides*), diacetoxyscirpenol (DAS) (*F. graminearum*, *F. cerealis*, *F. langsethiae*, *F. poae*, *F. sporotrichioides*, *F. acuminatum*, *F. equiseti*), monoacetoxyscirpenol (MAS) (*F. poae*, *F. sporotrichioides*, *F. equiseti*), neosolaniol (NEO) (*F. graminearum*, *F. langsethiae*, *F. sporotrichioides*), zearalenone (ZEA) (*F. graminearum*, *F. culmorum*, *F. cerealis*, *F. sporotrichioides*, *F. equiseti*), moniliformin (MON) (*F. avenaceum*, *F. tricinctum*, *F. acuminatum*), beauvericin (BEA) (*F. avenaceum*, *F. sporotrichioides*, *F. poae*), enniatins (ENN) (*F. avenaceum*, *F. tricinctum*, *F. poae*, *F. sporotrichioides*, *F. langsethiae*) [15,16,17,18,19,20,21,22].

*F. graminearum* and *F. culmorum* are considered the most virulent FHB causal agents. However, within individual species, different strains can exhibit significant variation in virulence [23,24,25,26] and toxin-producing potential [23,27,28,29,30,31,32]. Within species whose members typically produce specific mycotoxins, strains that are unable to produce mycotoxins or produce them at low levels have been reported [7,33,34]. The level of mycotoxin production may or may not correlate with the disease severity following FHB infection. Many studies have reported a positive correlation between the incidence and/or severity of the disease and mycotoxin concentration [35,36,37,38,39]. However, numerous other studies have found no correlation between mycotoxin concentration and infection severity [33,40,41], suggesting that mycotoxins may not be the primary factor driving grain infection. In some cases, high mycotoxin accumulation occurs even in the absence of visible FHB symptoms [42,43,44], posing an additional challenge for controlling grain quality and preventing the use of contaminated grain in production.

The composition of the FHB pathocomplex is influenced by geographic region (namely climatic characteristics), agronomic management, weather conditions, climate change, and presumably also by the host plant species. For example, in China, in northern regions characterized by drier and cooler conditions, *F. graminearum* was the predominant FHB pathogen, whereas in southern regions, which are wetter and warmer, *F. asiaticum* was more common [15]. Crop rotation has been shown to affect the chemotype composition of *F. asiaticum*: in maize–wheat rotation, 3-acetyldeoxynivalenol (3ADON) producers were prevalent, whereas in rice–wheat rotation, NIV producers were dominant [15]. Weather conditions in a given year, especially moisture levels during flowering, significantly influence the abundance of *F. graminearum* and its toxins, DON and ZEA, in wheat grain [15,45,46]. The confinement of particular FHB species to specific host plants cannot be considered a clearly established fact; however, a tendency toward host specificity has been discussed: *F. verticillioides* and *F. poae* more frequently parasitize maize and oats, respectively; *F. avenaceum* is considered more common in rye compared to other FHB species; and *F. equiseti* is more frequently isolated from barley than from other crops [47,48,49,50]. Thus, many factors may affect the composition of the FHB pathocomplex within a particular territory, which should be considered in disease management measures and mycotoxin control in agricultural production.

In Russia, the most comprehensive studies of the FHB pathocomplex have been conducted in the eastern regions (the Far East, Siberia, and the Ural region) and the north-western region (the Leningrad region). In these areas, the dominant species within the FHB pathocomplex are considered to be *F. graminearum*, *F. avenaceum*, *F. sporotrichioides*, and *F. poae* [51,52,53,54]. Additionally, several novel *Fusarium* species, such as *F. sibiricum* and *F. ussurianum*, have been identified in the Far East [55,56]. Herewith, *F. graminearum* has been shown to be represented by different chemotypes across Russia: in North Caucasian and North East populations, the 15-acetyl-deoxynivalenol (15ADON) and 3ADON chemotypes, respectively, dominate; meanwhile, in the Far East population, both chemotypes (15ADON and 3ADON) are present [21,53,55].

The species composition of spike-associated *Fusarium* pathocomplex in the Volga region (a key grain-producing area) has been considerably less studied. Individual strains from the Volga region have been characterized in terms of taxonomic affiliation and genetic features [57,58]; however, the phenotypes of most Volga strains, including virulence, remain undescribed, particularly with regard to field testing, which is generally conducted infrequently but serves as an important indicator of strain virulence and toxicity. Therefore, our study aimed to assess the species and intraspecific diversity of spike-associated *Fusarium* fungi characteristic of the Volga region, including their virulence and mycotoxin-producing potential.

## 2. Materials and Methods

### 2.1. Plant Sample Collection

**Figure 1:**
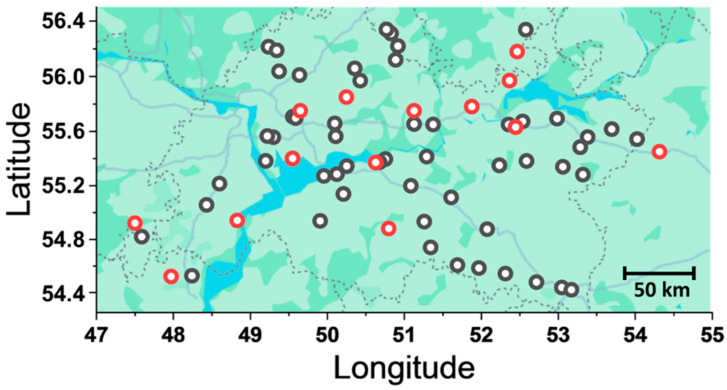
A map showing the locations where plant samples were harvested for the isolation of *Fusarium* strains. Red circles represent the locations from which *Fusarium* strains were isolated, whereas black circles represent the locations from which plant samples were taken but no *Fusarium* strains were isolated from these samples. Scale bar 50 km.

For the isolation of *Fusarium* fungi, plant samples—specifically spikes of wheat, rye, barley, and triticale—were collected from fields in the Volga region (Republics of Tatarstan, Bashkortostan, Chuvashia, and Udmurtia, as well as the Kirov, Orenburg, and Ulyanovsk regions) between mid-July to early August 2022 (the milky-waxy ripeness to full maturity stage, growth stage 75–92 [59]), while grain samples were obtained from grain stocks in December 2022. Samples were collected from 68 fields (commercial fields and variety testing sites, encompassing different cultivars of wheat, rye, triticale, and barley) and 15 grain storage facilities (Figure 1). In each field, 20 spikes were harvested approximately 30 m from the edge, at intervals of 30–40 m along the diagonal transect of the site. Samples were taken both from spikes with visible FHB symptoms (pink or orange coating, discoloration, necrotic darkening, streaking, ocular spotting of spikelet scales) and from those without visible symptoms. Samples were placed in sterile paper bags, transported to the laboratory, dried at 40 °C for 48 h, and stored at a cold temperature (4 °C) until use.

### 2.2. Isolation of Fusarium Fungi and Their Morphological Description

Parts of the cereal glumes were washed several times with water, surface-sterilized with 70% ethanol for 2–3 min, and then rinsed five times with sterile distilled water. Grains were washed several times with water, then sterilized sequentially with 1% SDS (twice for 10 min each), 0.01% potassium permanganate (for 10 min), and sodium hypochlorite (1% and 5%, each for 5 min), followed by five rinses with sterile distilled water. Sterilized plant samples under aseptic conditions were placed onto Petri dishes containing potato sucrose agar (1.5% of agar) (PSA) medium supplemented with tetracycline at a final concentration of 0.005%, and 0.4 µL/L Triton X-100 solution (Panreac, Barcelona, Spain). After 5–7 days of incubation in the dark at 25 °C, small mycelial fragments resembling those of *Fusarium* spp. were transferred to fresh PSA and cultured for an additional 5–7 days until conidia formed. Then, a single-spore isolate was obtained from each initially separated mycelium. Single-spore strains were grown on PSA for 5–7 days, and the morphology of their mycelium and conidia was analyzed using light microscopy («BIOMED-6» (BIOMED-SERVICE LLC, Moscow, Russia). Based on a set of morphological characteristics [60], the obtained strains were preliminarily assigned to specific *Fusarium* species.

### 2.3. The Analysis of DNA Sequences

The analyzed strains were grown for 7 days in liquid culture in potato sucrose medium (3% sucrose) (PSS). The mycelium was collected and homogenized using a Fast Prep-24 homogenizer (MP Biomedicals, Solon, OH, USA), and DNA was extracted using a DNeasy PowerSoil Pro Kit (Qiagen, Hilden, Germany) according to the manufacturer instructions. The quantity of extracted DNA was evaluated using NanoDrop 2000 spectrophotometer (Thermo Fisher Scientific, Waltham, MA, USA).

Fragments of the internal transcribed spacer 2 (ITS2) and the translation elongation factor 1-alpha (EF-1α) gene were PCR-amplified using Q5 Hot Start polymerase (NEB, Ipswich, MA, USA) and the primers ITS3_KYO2 (5′–GAT GAA GAA CGY AGY RAA–3′), ITS4 (5′–TCC TCC GCT TAT TGA TAT GC–3′) [61] and Fa_150 (5′–CCG GTC ACT TGA TCT ACC AG–3′) and Ra-2 (5′–ATG ACG GTG ACA TAG TAG CG–3′) [62], respectively. The amplification was performed under the following conditions: 98 °C for 2 min followed by 30 cycles of 98 °C for 15 s, 65 °C (for ITS2 amplification) or 52 °C (for EF-1α amplification) for 15 s, and 72 °C for 20 s; and a final stage of 72 °C for 5 min. The obtained PCR products were purified with AMPure XP magnetic beads (Beckman Coulter, Indianapolis, IN, USA) and then used for the preparation of DNA libraries according to the Illumina protocol (Illumina protocol, part no. 15044223, Rev. B). The indexing of libraries was performed using the Nextera XT Index Kit v2 (Illumina, San Diego, CA, USA), followed by purification of the indexed libraries with AMPure XP magnetic beads. The libraries were then pooled and sequenced on the MiSeq platform using the MiSeq Reagent Kit v3 (600 cycles) (Illumina, San Diego, CA, USA).

The obtained reads were processed for quality control and primer sequence removal using FastQC [63], MultiQC [64], and Cutadapt v.3.5 [65]. The DADA2 [66] pipeline was subsequently used for quality trimming, dereplication, chimera filtering, and the generation of amplicon sequence variants (ASVs). Taxonomic assignment of the generated ASVs was performed using NCBI BLAST (https://www.ncbi.nlm.nih.gov/ (accessed on 22 September 2025)) and the Fusarium multilocus sequence typing (MLST) database (https://fusarium.mycobank.org/page/Fusarium_identification (accessed on 22 September 2025)).

Based on the obtained sequences of ITS2 and EF-1α, the multigene phylogenetic analysis of the studied strains was performed. Along with the strains characterized in our study, previously characterized *Fusarium* spp. strains were included in the multigene phylogenetic analysis. The ITS2 and EF-1α nucleotide sequences for these strains were retrieved from the NCBI nucleotide database. A list of these strains, along with their sequence accession numbers, is presented in Supplementary File S1. For constructing the multigene phylogenetic tree, 293 bp fragments of ITS2 and 413 bp fragments of EF-1α were used. The multigene phylogenetic tree was constructed using the Neighbor-Joining method [67]. The percentages of replicate trees in which the associated taxa clustered together in the bootstrap test (10,000 replicates) are shown below the branches [68]. The evolutionary distances were computed using the Maximum Composite Likelihood method [69] and are expressed in units of the number of base substitutions per site. The analytical procedure encompassed 33 nucleotide sequences. The pairwise deletion option was applied to all ambiguous positions for each sequence pair, resulting in a final dataset comprising 706 positions. Evolutionary analyses were conducted in MEGA12 [70].

### 2.4. Virulence Assays

The virulence of the strains was assessed using two experimental approaches: under laboratory conditions and in the field.

#### 2.4.1. Laboratory Assay

The laboratory assay was conducted on aseptically grown winter rye plants (*Secale cereale* L. cv. Ogonek). Seeds were initially soaked in water at room temperature for 2 h, followed by treatment with a 5% AgNO_3_ solution for 15 min. After removing the AgNO_3_ solution, the seeds were washed five times with sterile 1% NaCl solution, each wash lasting 1 min. Finally, the seeds were rinsed with sterile water. The sterilized seeds were germinated in the dark at 23 °C for 2 days. Then, the seedlings were transferred to individual sterile 50 mL glass tubes containing 7 mL of diluted 1:4 Murashige-Skoog medium without organic carbon. The following day, seedlings were infected by placing an 8 mm diameter agar block, cut from the peripheral region of *Fusarium* strain colonies, into each test tube. Mock-infection was performed by placing sterile agar blocks into test tubes with seedlings. Each experimental variant was tested with at least 15 biological replicates. Plants were grown for 21 days at 20 °C with a 16 h light/8 h dark cycle photoperiod. Twenty-one days after infection, disease scores (necrotic lesions) were assessed and quantified using an 11-point scale, where 0 indicates no visible symptoms and 1–10 indicate that 10–100% of the seedling area is covered by necrotic lesions, respectively. Subsequently, plant biomass was harvested, and the dry weight of the roots was measured. The virulence of each strain was expressed both as the mean disease score and as the reduced root dry weight (RRDW, %) of infected plants compared to control plants since this parameter has been shown to allow more precise differentiation of strains based on their effect on the host plant than visual symptom assessment [71]; a greater reduction in root dry weight indicates higher strain virulence.

#### 2.4.2. Field Assay

Field assays of strain virulence were conducted on winter rye plants over a two-year period. In 2023, virulence was assessed on the cultivar Zilant, whereas in 2024, it was assessed on the cultivar Ogonek. Two different cultivars were used for the analysis of virulence due to their distinct immunological properties: cv. Zilant possesses moderate resistance to several plant diseases (rust, snow mold, and ergot) [72], whereas cv. Ogonek exhibits susceptibility to these diseases [73]. The experimental site was located in Bolshiye Kaban, Laishevo district (latitude 55.625164 N, longitude 49.351334 E), within the forest-steppe zone of the Volga region. Plants were grown in eight-row plots, each 1.5 m long with a row spacing of 0.15 m, and were treated with agrochemicals according to local recommendations for winter rye cultivation.

To produce inocula, conidial suspensions were prepared for each assayed strain. For this, an 8 mm diameter agar block, cut from the peripheral region of *Fusarium* strain colonies, was placed into 50 mL of SNB liquid medium, containing (g/L): KH_2_PO_4_—1; KNO_3_—1; MgSO_4_•7H_2_O—0.5; KCl—0.5; glucose—0.2; sucrose—0.2 [60]. Strains were grown at 25 °C, 150 rpm in the dark for 10 days. After 10 days of cultivation, the suspensions were filtered through five layers of sterile cheesecloth to remove mycelia. Conidia were then collected by centrifuging at 3000 rpm for 10 min and washed twice with sterile distilled water. Conidia concentrations were measured using a Goryaev counting chamber and adjusted to 1 × 10^5^ conidia/mL with sterile distilled water and 0.1% Tween-20 for plant inoculation.

Twenty spikes of similar height, selected from two randomized plots (10 plants per plot), were inoculated with each *Fusarium* strain. For plant infection, approximately 0.2 mL of conidia suspension (1 × 10^5^ conidia/mL) was injected with a syringe into both sides of the spikes at the central part during the beginning of the flowering stage, specifically phenological growth stage (GS) 59–61 [59]. Control spikes were inoculated with an equivalent volume of sterile distilled water. After inoculation, infected and mock-inoculated spikes were enclosed in transparent polythene bags to maintain high humidity; the bags were removed 72 h post-inoculation.

In field experiments, the virulence of the strains was assessed based on two parameters. First, virulence was quantified based on the severity of disease symptoms observed on spikes at full maturity stage using a 9-point scale: 1 = healthy spike, with scores from 2 to 9 corresponding to 0.5%, 10%, 30%, 50%, 70%, 80%, 90%, and 100% spike damage, respectively. Second, virulence was also quantified as the percentage reduction in grain weight of infected spikes compared to that of mock-infected spikes; a greater reduction indicated higher strain virulence. To determine the grain weight of infected and mock-infected spikes, the spikes were harvested manually at full maturity and threshed manually, and the grain from each spike was then weighed separately.

### 2.5. Detection of Mycotoxin-Related DNA Loci

Screening for the presence of genetic markers associated with mycotoxin production in the studied *Fusarium* strains was conducted using qPCR. Different mycotoxin-related genetic markers were determined depending on the species to which a strain belonged (Table 1). The genetic markers and corresponding primer sequences are listed in Table 1. qPCR was performed using the EvaGreen-containing master mix (Syntol, Moscow, Russia) according to the manufacturer’s instructions, and 5 ng (1 µL) of DNA was used as the template. Negative controls contained 1 µL of water instead of DNA sample.

PCR was performed under the following conditions: 95 °C for 2 min, followed by 35 cycles at 94 °C for 10 s, 50–65 °C (depending on the primer set) for 15 s, and 72 °C for 15–55 s. The reactions were run, and changes in fluorescence emission were detected using a CFX96 quantitative PCR system (Bio-Rad, Hercules, CA, USA). PCR products were analyzed by electrophoresis in 1% agarose gels.

### 2.6. Determination of Mycotoxins

Mycotoxins (deoxynivalenol (DON), zearalenone (ZEA), and T-2 toxin) were determined in both the grain used as a substrate for in vitro strain growth and the grain from artificially *Fusarium*-inoculated and mock-inoculated plants of winter rye cv. Ogonek, grown and artificially infected under field conditions as described in Section 2.4.2. For the determination of mycotoxins following in vitro strain growth, an 8 mm diameter agar block, cut from the peripheral region of *Fusarium* strain colonies, was placed onto 20 g of autoclaved wheat grains that had been hydrated overnight prior to autoclaving, and then cultured at 25 °C in the dark for 4 weeks. As a control, autoclaved wheat grains were maintained under the same conditions but without fungal inoculation. After 4 weeks, the grains were dried at 60 °C, ground to a fine powder, and used for mycotoxin analysis. For the determination of mycotoxins in grain of artificially infected and mock-infected spikes of plants of winter rye cv. Ogonek grown under field conditions, the spikes were harvested manually at full maturity and threshed manually. The cleaned grains were ground using a commercial laboratory mill equipped with a 1 mm sieve.

Mycotoxins were determined using ELISA kits from EVRICA Co., Ltd. (Moscow, Russia) according to the manufacturer’s instructions: DON (cat. No. 53621), ZEA (cat. No. 53620), and T-2 toxin (cat. No. 53624). The absorbance was measured at 450 nm using a CLARIOstar microplate reader (BMG Labtech GmbH, Ortenberg, Germany). Different mycotoxins were determined depending on the species to which a strain belonged: *F. graminearum*, *F. culmorum*–DON and ZEA, *F. sporotrichioides*–T-2 and ZEA, *F. poae*–T-2.

### 2.7. Statistics

To analyze the significance of differences in quantitative traits between different samples, the Mann–Whitney U test was used (with *p* < 0.05 indicating significance); for comparisons involving more than two samples, the Bonferroni correction for multiple comparisons was applied (with FDR < 0.05 indicating significance). Relationships between quantitative traits were assessed using Spearman’s rank correlation, with *p* < 0.05 considered indicative of a significant correlation. The statistical analysis was performed using OriginPro21 software (OriginPro, Version 2021, OriginLab Corporation, Northampton, MA, USA).

## 3. Results

### 3.1. Isolation of Fusarium ssp. Strains Inhabiting the Volga Region

Thirty-two candidate isolates of *Fusarium* spp. were isolated, and their single-spore strains were obtained. Based on the morphological characteristics of the strains (colony growth rate, abundance and color of aerial mycelium, presence of pigment, size and shape of conidiophores, micro- and macroconidia, chlamydospores, and mode of their formation [60]), they were preliminary assigned to the following species: *F. graminearum* (2 strains), *F. culmorum* (1 strain), *F. sporotrichioides* (15 strains), *F. avenaceum* (8 strains), and *F. poae* (6 strains) (File S2). Most of the strains from all species (22 strains) were isolated from wheat; six strains (five *F. sporotrichioides* and one *F. avenaceum*) were isolated from rye; and one and three strains of *F. sporotrichioides* were isolated from triticale and barley, respectively (File S2).

### 3.2. Genotyping and Phylogenetic Analysis of Fusarium ssp. Strains Inhabiting the Volga Region

**Figure 2:**
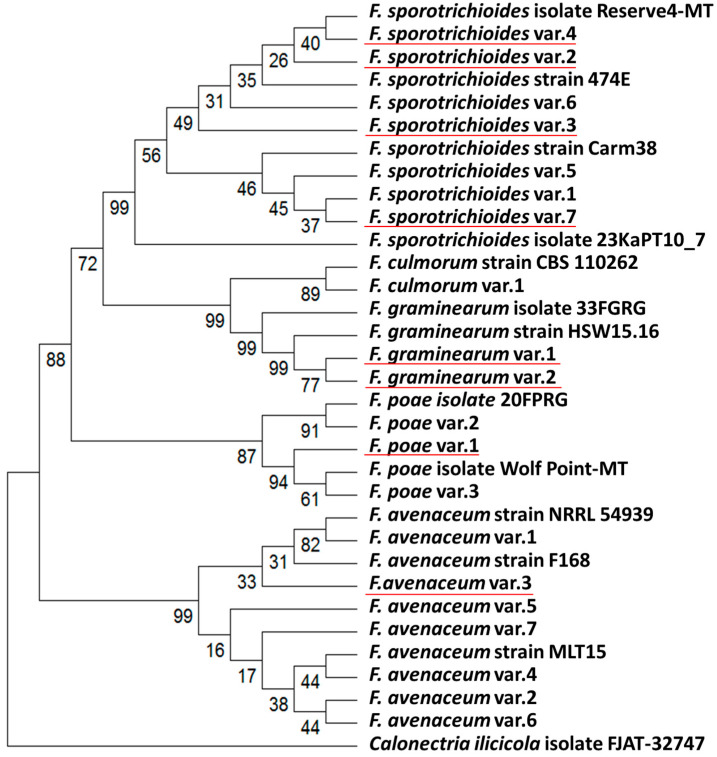
Multigene phylogenetic tree of *Fusarium* spp. strains isolated in this study and the closely related species *Calonectria ilicicola*, constructed based on sequences of the internal transcribed spacer 2 (ITS2) and a fragment of the elongation factor 1α gene (EF-1α). The phylogenetic tree was reconstructed using the maximum likelihood method with 10,000 bootstrap replicates, rooted at the midpoint, and visualized using MEGA 12. Bootstrap values are indicated below the nodes. Genotype variants (var.) within each species represent unique combinations of ITS2 and EF-1α sequences among the strains isolated in this study. Strains with the specified registration numbers were used as references because they represent those for which EF-1α and ITS2 sequences were previously sequenced and are available in the NCBI database (for details see File S1). Novel genotype variants identified in this study, characterized by ITS2 and EF-1α sequence combinations that do not match any strains in the Fusarium multilocus sequence typing (MLST) database or NCBI databases, are marked with a red line.

To verify the taxonomic classification of the studied *Fusarium* spp. strains and evaluate the interspecific relationships among the most prevalent species (*F. sporotrichioides*, *F. poae*, and *F. avenaceum*), the internal transcribed spacer 2 (ITS2) and translation elongation factor 1α (EF-1α) gene fragments were sequenced for each strain. The sequence analysis confirmed the species classification of the studied strains inferred from their morphological features (File S2). The analysis also revealed two novel EF-1α sequences, specifically sequences that were absent from current databases: one from *F. graminearum* (accession number PX380363) and one from *F. avenaceum* (accession number PX380355) (File S2). To assess the genetic similarity and variability among the isolated strains of *F. sporotrichioides*, *F. poae*, and *F. avenaceum*, a multigene phylogenetic tree was constructed (Figure 2). Based on the combination of ITS2 and EF-1α sequences within each strain, 20 genotype variants were identified among the 32 studied strains, including eight novel genotype variants whose ITS2 and EF-1α sequence combinations did not match any strains in the Fusarium multilocus sequence typing (MLST) database (https://www.fusarium.org/page/Poly%20ID%20Fusarium) or NCBI databases (accessed on 22 September 2025). These 20 genotype variants included: *F. sporotrichioides*—7 variants (including 4 novel), *F. poae*—3 variants (including 1 novel), *F. avenaceum*—7 variants (including 1 novel), *F. graminearum*—2 variants (both novel), and *F. culmorum*—1 variant. Previously analyzed strains with combinations of ITS2 and EF-1α sequences similar to those isolated in our study originated from Netherlands (*F. culmorum*, *F. avenaceum*), Belgium (*F. culmorum*), France (*F. sporotrichioides*), Canada (*F. poae*), China (*F. poae*, *F. avenaceum*), Finland and New Zealand (*F. avenaceum*).

### 3.3. Virulence of Fusarium ssp. Strains Inhabiting the Volga Region

**Figure 3:**
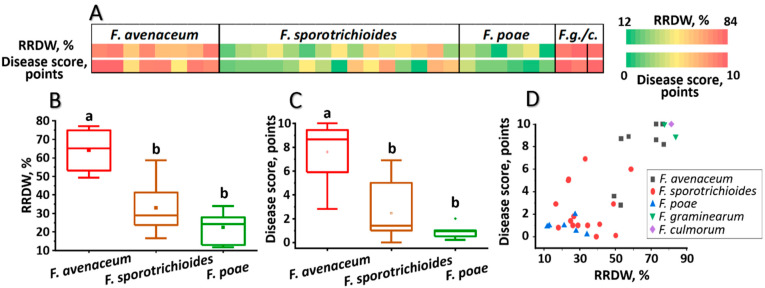
Virulence of *Fusarium* spp. strains inhabiting the Volga region toward aseptically grown winter rye cv. Ogonek. (**A**)—Heat map showing virulence-related parameters for each analyzed strain: (1) reduced root dry weight (RRDW, %) of infected plants compared to control plants and (2) disease score (points). *F.g/c*–*Fusarium graminearum* or *Fusarium culmorum*. (**B**,**C**)—Box plots showing the distribution of virulence-related parameters (RRDW (**B**) and disease score (**C**)) within the species *Fusarium avenaceum*, *Fusarium sporotrichioides*, and *Fusarium poae*. Different letters above the bars indicate significant differences between species (Mann–Whitney test with Bonferroni correction for multiple comparisons, FDR < 0.05). (**D**)—Scatter plot showing the spatial distribution of strains based on two virulence-related parameters: disease score and RRDW; *F. culmorum*, *F. graminearum*, *F. avenaceum*, *F. sporotrichioides*, and *F. poae* are indicated by purple, green, black, red, and blue figures, respectively (see legend on the plot).

Virulence of the analyzed *Fusarium* spp. strains toward winter rye was assayed under both laboratory and field conditions. Under laboratory conditions, virulence was evaluated toward cv. Ogonek and expressed by two parameters: the level of disease lesions caused by the strain (disease score, points) and the reduced root dry weight (RRDW, %) of infected plants compared to control plants. *F. graminearum* (two strains) and *F. culmorum* (one strain) exhibited high virulence, with all three strains causing a disease score of 9–10 points (on an 11-point scale) and RRDW of 78–84% (Figure 3, File S2). Within each of the other three species, these parameters varied significantly: *F. avenaceum*—3–10 points and 49–77% RRDW, *F. sporotrichioides*—0–7 points and 17–59% RRDW, *F. poae*—0–7 points and 12–34% RRDW (Figure 3, File S2). On average, among these three species, *F. avenaceum* was the most virulent. *F. poae*, based on the meanings of virulence indicators, tended to be the least virulent; however, these values did not significantly differ from those for *F. sporotrichioides* (Figure 3).

**Figure 4:**
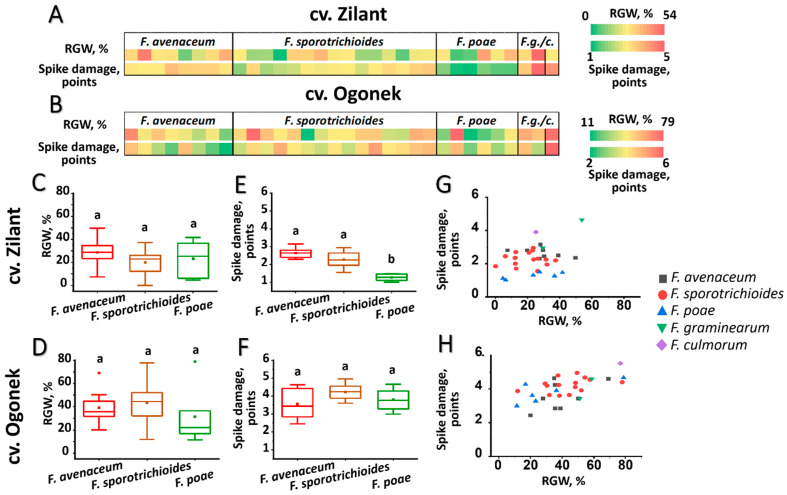
Virulence of *Fusarium* spp. strains inhabiting the Volga region toward field grown winter rye: more resistant cultivar Zilant (**A**,**C**,**E**,**G**) and more susceptible cultivar Ogonek (**B**,**D**,**F**,**H**). (**A**,**B**)—Heat maps showing virulence-related parameters for each analyzed strain: (1) reduced grain weight (RGW, %) of infected spikes compared to that of mock-infected spikes and (2) spike damage (points). *F.g/c*–*Fusarium graminearum* or *Fusarium culmorum*. (**C**,**F**)—Box plots showing the distribution of virulence-related parameters (RGW (**C**,**D**) and spike damage (**E**,**F**)) within the species *Fusarium avenaceum*, *Fusarium sporotrichioides*, and *Fusarium poae*. Different letters above the bars indicate significant differences between species (Mann–Whitney test with Bonferroni correction for multiple comparisons, FDR < 0.05). (**G**,**H**)—Scatter plots showing the spatial distribution of strains based on two virulence-related parameters: spike damage and RGW; *F. culmorum*, *F. graminearum*, *F. avenaceum*, *F. sporotrichioides*, and *F. poae* are indicated by purple, green, black, red, and blue figures, respectively (see legend on the plot).

**Figure 5:**
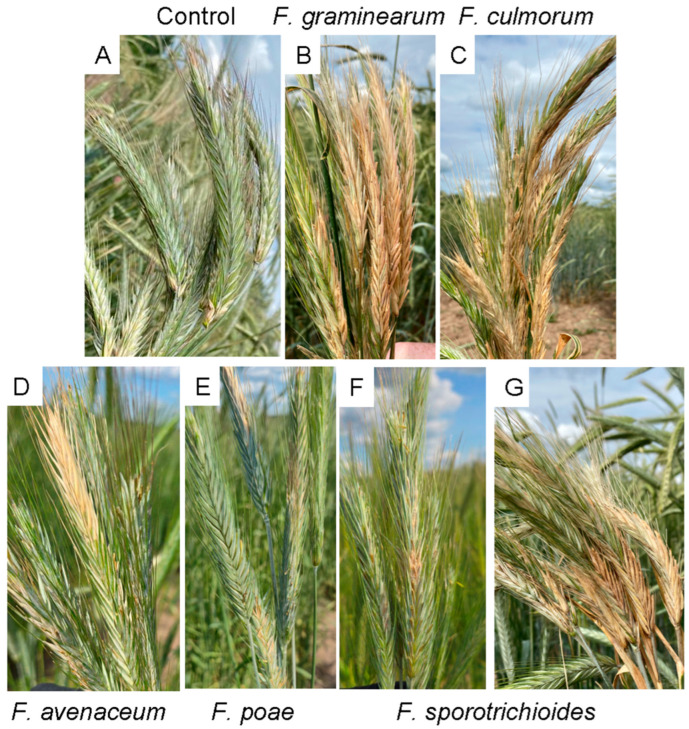
Spikes of winter rye cultivar Ogonek at the medium milk to early dough development stage (phenological growth stage (GS) 75–83 [59]). Spikes were mock-inoculated (**A**) or inoculated with a highly virulent *Fusarium graminearum* strain (**B**), a highly virulent *Fusarium culmorum* strain (**C**), a moderately virulent *Fusarium avenaceum* strain (**D**), a low-virulent *Fusarium poae* strain (**E**), and low-virulent (**F**) and highly virulent (**G**) strains of *Fusarium sporotrichioides*.

In field experiments, the *F. culmorum* strain (the only strain of this species among the 32 strains analyzed in this study) was among the most virulent toward the more susceptible cultivar Ogonek, causing a 77% reduction in grain weight (RGW) from the spike and a disease score of 6 (Figure 4 and Figure 5, File S2). However, its virulence toward the more resistant cultivar Zilant was lower than that toward cv. Ogonek, with a reduction in grain weight of ~25%. Two *F. graminearum* strains also exhibited high virulence toward cv. Ogonek (RGW 51–59%, disease score 3–5), with one of them demonstrating a similarly high level of virulence toward cv. Zilant (RGW 54%, disease score 5) (Figure 4 and Figure 5, File S2).

The three most represented species (*F. avenaceum*, *F. sporotrichioides*, and *F. poae*) exhibited high intraspecific between-strain variability in virulence-related traits; however, on average, each species caused a similar reduction in grain weight from the spike–approximately 30% in more resistant cv. Zilant and 40% in more susceptible cv. Ogonek (Figure 4, File S2).

No significant differences in the disease scores were observed among the three species, except that *F. poae* caused significantly fewer disease symptoms in the more resistant cv. Zilant and exhibited smaller between-strain variability in this trait compared to *F. avenaceum* and *F. sporotrichioides* (Figure 4, File S2). Herewith, disease scores in the more resistant cv. Zilant were lower than those in the more susceptible cv. Ogonek. Regarding the two virulence-related parameters assessed in the cv. Zilant (RGW and disease score), *F. poae* strains clustered separately from the majority of strains of the other two species, indicating the relatively low virulence of *F. poae* (Figure 4). Although *F. avenaceum*, *F. sporotrichioides*, and especially *F. poae* displayed lower average virulence compared to the assayed strains of *F. culmorum* and *F. graminearum*, individual strains within these species (*F. sporotrichioides* FsM10005, *F. avenaceum* FsM10014, and *F. poae* FsM10007) exhibited very high virulence toward the more susceptible cv. Ogonek (but not toward the more resistant cv. Zilant), reducing grain weight from the spike by up to 70–80% and resulting in disease scores of 4–5 (File S2).

### 3.4. Mycotoxin-Producing Potential of Fusarium ssp. Strains Inhabiting the Volga Region

The mycotoxin-producing potential of the analyzed *Fusarium* spp. strains was assessed using the following three approaches: (1) detection of genetic markers via PCR; (2) determination of toxins using ELISA in fungal cultures grown in vitro on autoclaved wheat grain; and (3) quantification of toxins in the grain of field-grown rye plants (cv. Ogonek) artificially inoculated with the tested strains. *F. culmorum* and *F. graminearum* strains were found to possess the trichothecene genotype: specifically, the single *F. culmorum* strain and one of the two *F. graminearum* strains (FsM10048) possessed the 3ADON chemotype, whereas the other *F. graminearum* strain (FsM10030) possessed the NIV chemotype (File S2). The PCR analysis of mycotoxin-related markers was consistent with the results of mycotoxin determination by ELISA: the *F. culmorum* strain and *F. graminearum* FsM10048 (but not FsM10030) produced high levels of DON both in vitro on autoclaved grain (File S2) and in the grain of artificially inoculated, field-grown rye (Table 2). Additionally, the analyzed *F. culmorum* and *F. graminearum* strains produced high levels of zearalenone (ZEA) in vitro on autoclaved grain (File S2). However, in infected spikes of field-grown plants, *F. culmorum* produced only small amounts of ZEA, around the maximum permissible concentration, whereas *F. graminearum* strains did not produce ZEA in the infected spikes (Table 2).

The studied *F. poae* strains exhibited the NIV genotype. Most of the *F. sporotrichioides* strains exhibited a ZEA-negative genotype and did not produce ZEA when grown in vitro on autoclaved grain (File S2). However, four of the fifteen *F. sporotrichioides* strains (FsM10031, FsM10053, FsM10055, and FsM10056) possessed the ZEA genotype and produced ZEA in vitro on autoclaved grain (File S2). Despite this, the ZEA levels in grains from field-grown plants infected by these ZEA-producing *F. sporotrichioides* strains were either below or only slightly above the detection threshold, significantly below the maximum permissible concentration, and only about twofold higher than in non-inoculated grains with a background level of natural mycotoxin contamination (Table 2). All analyzed *F. sporotrichioides* strains also exhibited trichothecene genotype and T-2 genotype and produced high levels of T-2 toxin when grown in vitro on autoclaved grain (File S2). The grain of field-grown plants inoculated with these strains was also heavily contaminated with T-2 toxin, with levels ranging from 18 to 37 mg/kg (Table 2). All analyzed *F. avenaceum*, *F. poae*, and *F. sporotrichioides* strains exhibited enniatin genotype (File S2).

### 3.5. Analysis of Potential Relationships Between Various Virulence Parameters of Fusarium ssp. Strains Inhabiting the Volga Region

**Figure 6:**
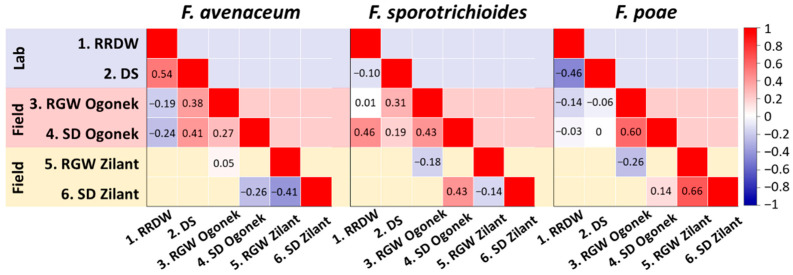
Spearman’s rank correlation coefficients between six virulence-related parameters of *Fusarium avenaceum*, *Fusarium poae*, and *Fusarium sporotrichioides* strains inhabiting the Volga region. Virulence was assessed under laboratory conditions on a more susceptible cultivar Ogonek (blue background) and under field conditions on a more susceptible cultivar Ogonek (pink background) and a more resistant cultivar Zilant (yellow background). Virulence was expressed as: (1) reduced root dry weight (RRDW, %) of aseptically grown inoculated plants of cv. Ogonek compared to control non-inoculated plants; (2) the level of disease lesions caused by the strain in aseptically grown cv. Ogonek (disease score, points); (3) reduction in grain weight (RGW, %) per spike in inoculated field-grown cv. Ogonek compared to control non-inoculated plants; (4) the level of disease lesions on the spikes of field-grown cv. Ogonek (spike damage, points); (5) reduction in grain weight (RGW, %) per spike in inoculated field-grown cv. Zilant compared to control non-inoculated plants; (6) the level of disease lesions on the spikes of field-grown cv. Zilant (spike damage, points). The statistical significance of the presented correlation coefficients was above 0.05.

The potential relationships between various virulence parameters of the studied strains were assessed separately for the three most represented species (*F. avenaceum*, *F. poae*, and *F. sporotrichioides*). Two virulence parameters evaluated under laboratory conditions (RRDW and disease score) showed a moderate positive correlation in the most virulent of the three species, *F. avenaceum*, but not in *F. poae* and *F. sporotrichioides* (Figure 6). In the least virulent species, *F. poae*, a slight negative correlation was observed between these two parameters. No correlation was observed between virulence parameters assessed under laboratory and field conditions in the more susceptible cv. Ogonek within the least virulent species, *F. poae*. However, some field-assessed and laboratory-assessed parameters showed a slight positive correlation within the species *F. avenaceum* and *F. sporotrichioides* (Figure 6).

Within *F. poae*, a moderate positive correlation was observed between the two virulence parameters evaluated under field conditions—RGW and spike damage—on both cultivars (Figure 6). Within *F. sporotrichioides*, a slight positive correlation between these parameters was observed only in the more susceptible cv. Ogonek, whereas within *F. avenaceum*, a slight negative correlation was observed when these parameters were analyzed on the more resistant cv. Zilant. No correlation was observed between the two cultivars in either RGW or spike damage caused by the studied *Fusarium* species, except for a slight positive correlation in spike damage caused by *F. sporotrichioides* strains between the two cultivars (Figure 6). Due to the small sample size and high deviation in some samples, the data did not fulfill the requirements for normality and equal variances. Therefore, the relationships described above were characterized by statistical significance above 0.05 and can only be interpreted as indicative.

No relationships were found between the ZEA-producing ability of the *F. sporotrichioides* strains (4 strains produced ZEA, while 11 did not) and any of the six analyzed virulence-related parameters (File S3). Similarly, no significant correlation was observed between the levels of T-2 toxin in grains from artificially *F. sporotrichioides*-inoculated plants under field conditions and the six analyzed virulence-related parameters (File S4).

## 4. Discussion

In this study, we aimed to gain a deeper insight into the species and intraspecific diversity of *Fusarium* fungi associated with the spikes of cereal crops cultivated in the Volga region. Thirty-two strains were isolated from the spikes of field-grown cereal crops and from grain stocks. Most of the strains belonged to the species *F. sporotrichioides*, *F. poae*, and *F. avenaceum*, which aligns with the high frequency of these species in Russia [58]. Due to their high ecological plasticity, these fungal species are less demanding in terms of warmth and humidity compared to *F. culmorum* and *F. graminearum*, enabling them to thrive in a moderate climate [79,80]. In addition, one *F. culmorum* strain and two *F. graminearum* strains were isolated in our study. *F. graminearum*, which is considered the dominant causal agent of FHB in regions with warm and wet climates, has been shown to be widely represented in the Far East and southern parts of Russia [21,53]. This species has also been detected in the north-west part of Russia. *F. culmorum* has been frequently found in grains from the central and north-west parts of Russia; however, its frequency of occurrence in Russia has been reported to be decreasing [21]. The 32 isolated strains were grouped into 20 genotypes, defined as combinations of ITS2 and EF-1α sequences. Almost half of the identified genotypes (8 out of 20) were unique, meaning their ITS2 and EF-1α sequence combinations did not match any strains in the MLST or NCBI databases. This presumably indicates that the structure of the FHB pathocomplex in the Volga region has distinctive features.

*F. culmorum* and *F. graminearum* are considered the most virulent and devastating FHB pathogens [29]. In our study, strains of these two species were also among the most virulent when assessed under both laboratory and field conditions. However, these two species were poorly represented in the sample of isolated strains, indicating that they are likely to be rather rare in the Volga region. *F. avenaceum*, *F. sporotrichioides*, and *F. poae*, which were the most prevalent within the studied sample of strains, are considered relatively weak pathogens, especially the latter two, compared to *F. culmorum* and *F. graminearum* [81,82,83]. Indeed, in our study, the average virulence of strains of each of these three species was lower than that of strains of *F. culmorum* and *F. graminearum*. Herewith, a very high intraspecific variation in virulence was observed among these three species, with different strains within each species exhibiting a range of virulence levels from very low to very high. High intraspecific variability has previously been shown for the most studied FHB pathogens, *F. culmorum*, *F. graminearum*, and *F. avenaceum*, with different strains exhibiting varying levels of virulence and toxin-producing potential [25,84,85,86]. However, intraspecific variability in *F. sporotrichioides* and *F. poae* remains largely uncharacterized. Previously compared strains of *F. poae* species exhibited varying degrees of symptom severity on spikes; however, none caused severe symptoms or reduced crop yield [33]. In contrast, in our study, we identified strains of *F. poae* and *F. sporotrichioides* with very high virulence levels (comparable to those of *F. culmorum* and *F. graminearum*), indicating that some representatives of these species pose a serious threat to grain production.

The virulence potential of the studied strains was often expressed differentially depending on the specific parameter assessed and the type of virulence assay. Among the most prevalent species (*F. poae*, *F. avenaceum*, and *F. sporotrichioides*), a moderate positive correlation between the two parameters evaluated under laboratory conditions (RRDW and disease score) was observed only within the most virulent species, *F. avenaceum*, whereas within the least virulent species, the correlation was negative. In contrast, under field conditions, a moderate positive correlation between the two assessed virulence parameters (RGW and spike damage) was observed only in the least virulent species, *F. poae*. In previous studies, an association between the severity of FHB symptoms and plant productivity was observed in plants inoculated with more virulent, but not less virulent, *Fusarium* species [83,87]. No correlation was observed between the two cultivars in terms of both RGW and spike damage caused by the studied strains, except for a slight positive correlation between the spike damage caused by *F. sporotrichioides* in the two cultivars. This means that the variety of the crop host determines the hierarchy of strains in terms of their virulence levels. Thus, to gain deeper insights into the characteristics of *Fusarium* species and strains, alternative experimental models should be applied. In our study, screening strains for their virulence on a more resistant cultivar (Zilant) enabled more precise differentiation between species based on this criterion, while screening on a more susceptible cultivar (Ogonek) facilitated clearer differentiation between various strains within a single species and demonstrated the intraspecific variability of these species in terms of virulence. Specifically, *F. poae* exhibited significantly reduced virulence compared to *F. avenaceum* and *F. sporotrichioides* only when screened on the cv. Zilant. In contrast, screening on the cv. Ogonek revealed high intraspecific variation in RGW for *F. sporotrichioides*, *F. avenaceum*, and *F. poae*, with ranges of 12–78%, 20–69%, and 11–79%, respectively.

In terms of mycotoxin-producing potential, the studied *F. culmorum* strain exhibited the 3ADON chemotype and produced DON under both in vitro and field conditions (with levels around 30 times higher than the maximum permissible concentration), which aligns with the fact that all described strains of this species originating from Russia possess the 3ADON chemotype [88]. Similarly, one of the studied *F. graminearum* strains exhibited the 3ADON chemotype, whereas the other possessed the NIV chemotype and did not produce DON. To the best of our knowledge, *F. graminearum* strains with the NIV chemotype have not been previously described in Russia, where only the DON chemotype of this species has been identified: 15ADON in the North Caucasian population, 3ADON in the North West part, and both 3ADON and 15ADON in the Far East [88,89]. The isolated DON-producing *F. graminearum* strain displayed higher virulence toward the more resistant cultivar Zilant under field conditions than the DON-non-producing strain. This is consistent with the well-established role of DON in virulence and with the observation that DON-producing *F. graminearum* and *F. culmorum* strains display higher virulence than NIV-producing strains [27,90].

All studied *F. culmorum* and *F. graminearum* strains produced zearalenone in vitro. However, a baseline level of zearalenone (typical of control, non-infected plants with background levels of natural mycotoxin contamination, which are well below the maximum permissible concentrations in Russia and around the maximum permissible concentrations in EU) was detected in the grains of *F. graminearum*-inoculated rye plants grown under field conditions, and only a low level of zearalenone (around the maximum permissible concentration in Russia) was observed in the grains of *F. culmorum*-inoculated plants. Despite the potential ability to synthesize various mycotoxins, it has been widely demonstrated that different *Fusarium* species often do not express this capability, which depends on a range of environmental factors [20,27,91,92]. Specifically, *F. culmorum* and *F. graminearum* strains that can synthesize high amounts of zearalenone in vitro have previously been shown to produce little or no zearalenone when colonizing plants under field conditions [20,93], which is consistent with the results obtained in our study.

All fifteen studied *F. sporotrichioides* strains produced T-2 toxin at high levels both in vitro and in the grains of field-grown plants (roughly ~400 times higher than the maximum permissible concentration). Previously, *F. sporotrichioides* strains have also been shown to synthesize high levels of T-2 toxin, with the levels differing by more than 40-fold depending on the particular *F. sporotrichioides* strain [94]. In our study, only around 2-fold differences in T-2 toxin levels were observed in grains of field-grown rye plants following infection with the studied *F. sporotrichioides* strains, and no relationships between T-2 toxin level and virulence were found. This is consistent with a previous study showing no association between the percentage of symptomatic kernels and T-2 and HT-2 content in *F. sporotrichioides*-infected wheat [87].

Our results clearly show that even nearly asymptomatic infection caused by *F. sporotrichioides* strains with low virulence is associated with the accumulation of high levels of T-2 toxin in grains of infected field-grown plants comparable to that caused by highly virulent strains of this species. Previously, high levels of *Fusarium* mycotoxins have also been detected in visually asymptomatic plants, indicating that apparently intact grain may pose a health risk if consumed and highlighting the need for rigorous mycotoxin monitoring in agricultural production [42,44,95].

In contrast to their similar T-2 toxin-producing potential, the studied *F. sporotrichioides* strains differed markedly in their ability to synthesize zearalenone: four of the 15 strains of this species possessed the zearalenone genotype and produced zearalenone in vitro, whereas the remaining 11 strains exhibited zearalenone-negative genotype and phenotype. To the best of our knowledge, such differentiation among *F. sporotrichioides* strains based on their ability to produce zearalenone has not been previously reported. The ability to produce zearalenone was not associated with the virulence of the studied *F. sporotrichioides* strains: zearalenone-positive and zearalenone-negative strains did not differ in virulence. This is in accordance with zearalenone being considered not to exhibit phytotoxic properties and not to play a role in the virulence of zearalenone-producing fungi [96,97]. This also aligns with our results showing that zearalenone-producing potential of *F. sporotrichioides* was not expressed under field conditions: the levels of zearalenone in grains of inoculated plants were either below or only slightly above the detection threshold and only about twofold higher than in non-inoculated grains with a background level of natural mycotoxin contamination (which are well below the maximum permissible concentrations in Russia and around the maximum permissible concentrations in EU). A similar pattern of zearalenone production, where *Fusarium* strains produce this mycotoxin at high levels in vitro but do not synthesize it (or synthesize it at very low levels) in infected grain, has been previously reported for species such as *F. culmorum* and *F. graminearum* [20,98].

## 5. Conclusions

Among the *Fusarium* spp. strains isolated from cereal crops in the Volga region, the species *F. sporotrichioides*, *F. poae*, and *F. avenaceum* were predominant. Among these strains, nearly half exhibited unique genotypes, meaning that their ITS2 and EF-1α sequence combinations did not match those of any strains in the MLST or NCBI databases. The virulence potential of most of the studied strains was expressed differentially depending on the specific parameter assessed and the type of virulence assay. High intraspecific variability in virulence was observed for the first time within *F. sporotrichioides* and *F. poae* species, and highly virulent strains were identified for the first time within these species. Not only symptomatic but also asymptomatic (weakly expressed) infections caused by *F. sporotrichioides* were shown to be associated with the accumulation of high levels of T-2 toxin in the grains of infected plants. *F. sporotrichioides* strains were first demonstrated to exhibit intraspecific variability in zearalenone-producing potential. A *F. graminearum* strain possessing the nivalenol chemotype was first identified in Russia; this strain displayed lower virulence toward the more resistant rye cultivar under field conditions than the deoxynivalenol-producing *F. graminearum* strain.

## Figures and Tables

**Table 1:** Primers used for screening mycotoxin-related genetic markers in the studied *Fusarium* strains. 3-acetyl-deoxynivalenol (3ADON), 15-acetyl-deoxynivalenol (15ADON), nivalenol (NIV), T-2 toxin (T-2), zearalenone (ZEA), enniatins (ENN).

Toxin Type	Gene	Forward Primer (5′-3′)	Reverse Primer (5′-3′)	Tested Species	Reference
Trichotecene	*tri5*	CAGATGGAGAACTGGATGGT	GCACAAGTGCCACGTGAC	*F. graminearum*, *F. culmorum*	[74]
3ADON	*tri12*	AACATGATCGGTGAGGTATCGA	CCATGGCGCTGGGAGTT	*F. graminearum*, *F. culmorum*	[75]
15ADON	*tri12*	GTTTCGATATTCATTGGAAAGCTAC	CAAATAAGTATCGTCTGAAATTGGAAA		
NIV	*tri12*	GCCCATATTCGCGACAATGT	GGCGAACTGATGAGTAACAAAACC		
NIV	*tri7*	TATCCTTGCATGGCAATGCC	AAATGGCGATACGAGTATTGA	*F. poae*	[76]
T-2	*tri16*	GGTGAGATTGCTTCGATGTG	CTCAAAGGGCGAATCAACTAC	*F. sporotrichioides*, *F. poae*	This study
ZEA	*PKS4*	CGTCTTCGAGAAGATGACAT	TGTTCTGCAAGCACTCCGA	*F. graminearum*, *F. culmorum* *F. sporotrichioides*	[77]
ENN	*esyn1*	GGTCTCGATCCATCCAAGTC	GTGAAGAAGGCAGGCTCAAC	*F. avenaceum*	[78]
		GGCCTTGAGCCATCCAGATC	CTCGTTGGTAGCCTGCGATCG	*F. poae*, *F. sporotrichioides*	

**Table 2:** Mycotoxin content (µg/kg) in grains from field plot-grown winter rye Ogonek, artificially inoculated with studied strains of *Fusarium sporotrichioides*, *Fusarium graminearum*, and *Fusarium culmorum*. DON—deoxynivalenol; ZEA—zearalenone; T-2—T-2 toxin. N/A—not analyzed.

Strain	Mycotoxin		
	DON	T-2	ZEA
*F. sporotrichioides* 10001	N/A	23,799.8	135.6
*F. sporotrichioides* 10005	N/A	36,627.7	151.9
*F. sporotrichioides* 10011	N/A	25,988.7	303.9
*F. sporotrichioides* 10012	N/A	32,668.9	165.8
*F. sporotrichioides* 10031	N/A	33,542.7	0.0
*F. sporotrichioides* 10034	N/A	32,668.9	196.6
*F. sporotrichioides* 10035	N/A	27,883.9	0.0
*F. sporotrichioides* 10039	N/A	27,397.5	0.0
*F. sporotrichioides* 10046	N/A	17,646.3	146.9
*F. sporotrichioides* 10051	N/A	22,378.2	236.7
*F. sporotrichioides* 10053	N/A	28,882.7	264.4
*F. sporotrichioides* 10055	N/A	28,130.3	0.0
*F. sporotrichioides* 10056	N/A	33,839.1	0.0
*F. sporotrichioides* 10057	N/A	27,397.5	0.0
*F. sporotrichioides* 10058	N/A	32,668.9	0.0
*F. graminearum* 10030	N/A	N/A	62.6
*F. graminearum* 10048	260,811.9	N/A	84.3
*F. culmorum* 10028	302,257.6	N/A	918.4
Control mock-inoculated grain	730.7	4394.5	119.4
Detection limit	100.0	24.0	100.0
Maximum permissible concentrations			
Russia	700.0	100.0	1000.0
EU	1000.0	50.0	100.0

## Data Availability

The described DNA sequences are available at the National Center for Biotechnology Information (NCBI) (accession numbers for the ITS2 sequences: PX363249-PX363280; for the EF-1α sequences: PX380334-PX380365).

## Supplementary Materials

The following supporting information can be downloaded at: https://www.mdpi.com/article/10.3390/jof11120841/s1, File S1: Accession numbers for the internal transcribed spacer 2 (ITS2) and elongation factor 1α gene (EF-1α) sequences of *Fusarium* species (with *Calonectria ilicicola* as the outgroup) used in the multigene phylogenetic analysis; File S2: Dataset showing the origin and characteristics of the analyzed *Fusarium* spp. strains; File S3: Association between zearalenone synthesis and virulence of *F. sporotrichioides* strains; File S4: The spearman’s correlation between the levels of T-2 toxin in grains of plants artificially inoculated with *F. sporotrichioides* under field conditions and the six analyzed virulence-related parameters of *F. sporotrichioides*.
